# Intravenous Fosfomycin for Systemic Multidrug-Resistant *Pseudomonas aeruginosa* Infections

**DOI:** 10.3390/antibiotics12121653

**Published:** 2023-11-23

**Authors:** Giuseppe Pipitone, Stefano Di Bella, Alberto Enrico Maraolo, Guido Granata, Milo Gatti, Luigi Principe, Alessandro Russo, Andrea Gizzi, Rita Pallone, Antonio Cascio, Chiara Iaria

**Affiliations:** 1 Infectious Diseases Unit, ARNAS Civico-Di Cristina Hospital, 90127 Palermo, Italy; pep2pe@gmail.com (G.P.);; 2 Clinical Department of Medical, Surgical and Health Sciences, Trieste University, 34127 Trieste, Italy; 3 First Division of Infectious Diseases, Cotugno Hospital, Azienda Ospedaliera dei Colli, 80131 Naples, Italy; 4 Clinical and Research Department for Infectious Diseases, National Institute for Infectious Diseases L. Spallanzani, IRCCS, 00149 Rome, Italy; 5 Department of Medical and Surgical Sciences, University of Bologna, 40138 Bologna, Italy; 6 Clinical Pharmacology Unit, IRCCS University Hospital of Bologna, 40138 Bologna, Italy; 7 Microbiology and Virology Unit, Great Metropolitan Hospital “Bianchi-Melacrino-Morelli”, 89133 Reggio Calabria, Italy; 8 Infectious and Tropical Diseases Unit, Department of Medical and Surgical Sciences, ‘Magna Graecia’ University of Catanzaro, 88100 Catanzaro, Italy; 9 Infectious Diseases Unit, University Hospital P. Giaccone, 90127 Palermo, Italy; 10 Infectious and Tropical Diseases Unit, University Hospital “Renato Dulbecco”, 88100 Catanzaro, Italy

**Keywords:** fosfomycin, *Pseudomonas aeruginosa*, multidrug resistance, difficult-to-treat *P. aeruginosa*

## Abstract

Human *Pseudomonas* infections have high morbidity and mortality rates. *Pseudomonas* bacteria can cause sepsis or septic shock; they produce biofilm and commonly exhibit a multidrug-resistant phenotype. The choice of antimicrobial therapy in many cases is challenging, and deep knowledge of clinical, microbiological, and pharmacological issues is required. Intravenous fosfomycin is being repurposed in a combination given its favorable pharmacokinetic/pharmacodynamic properties (a small molecule with favorable kinetic both in bloodstream infection and in deep-seated infections), antibiofilm activity, and its interesting synergistic effects with other antimicrobials. Recent literature on epidemiological, microbiological, pharmacological, and clinical data on intravenous fosfomycin therapy against *Pseudomonas* is herein reviewed and discussed.

## 1. Introduction

Infections caused by *Pseudomonas* have high mortality rates, especially in patients with hematologic/oncologic underlying diseases. These mortality rates are motivated by the commonly underlying hematologic/oncologic diseases in *Pseudomonas*-infected patients, the unique pathogenicity of the bacterium and its toxins, and the common multidrug-resistant (MDR) phenotype, especially in nosocomial strains. For decades, the debate has revolved around whether combination therapy is superior to monotherapy for *Pseudomonas* infections. Usually, combination therapy is not recommended, although it is considered in some instances [1]. Intravenous fosfomycin is an increasingly used “old” drug. Its benefits arise from both pharmacokinetic/pharmacodynamic (PK/PD) properties and synergistic properties with other antibiotics [2], which allow for the reduction of microorganisms’ minimal inhibitory concentrations (MICs), and, in some cases, even restore susceptibility to resistant strains [3]. In addition, fosfomycin is known to retain good antibiofilm activity against both Gram-positive and Gram-negative bacteria (including *Pseudomonas*) [4], and this property may be exploited in cystic fibrosis and infected ulcers/wounds. The European Committee on Antimicrobial Susceptibility Testing (EUCAST) does not provide clinical breakpoints for intravenous fosfomycin toward *Pseudomonas*. However, the epidemiological cut-off value (ECOFF) is 256 mg/L [5]. Despite this seemingly unfavorable antimicrobial in vitro activity, in an analogy to what has been observed for *Acinetobacter*, there are emerging pieces of evidence regarding the usefulness of intravenous fosfomycin in infections caused by *Pseudomonas*, although most of them still have mainly microbiological or PK/PD outcomes. Intravenous fosfomycin should always be administered as part of a combination regimen for *Pseudomonas* infections since heteroresistance is extremely common [6]. In this paper, we review the existing updated evidence of microbiological, pharmacological, and clinical data on the use of intravenous fosfomycin for *Pseudomonas aeruginosa* human infections.

## 2. Results

### 2.1. Microbiology

#### 2.1.1. Testing

Fosfomycin susceptibility testing presents several challenges. The traditional agar dilution technique in the presence of glucose-6-phosphate (G6P) is considered by both EUCAST and the Clinical & Laboratory Standards Institute (CLSI) the reference method for fosfomycin susceptibility testing. The addition of G6P allows the entry of fosfomycin into bacterial cells, resulting in a lower MIC value. However, the traditional method is not often performed due to its labor- and time-intensive characteristics. In fact, in our review, showed in Table 1 [7,8,9,10,11,12,13,14,15,16,17,18,19,20,21,22,23,24,25,26,27,28], we found 22 articles regarding in vitro (n = 20) and in vivo (n = 2) studies on the antimicrobial activity of fosfomycin (alone or in combination); the traditional method was used in 1093 (75.6%) out of a total of 1445 *P. aeruginosa* isolates, other methods were used in 267 (18.5%): such as broth microdilution (checkerboard in the case of antibiotic combination) and Etest (alone and in combination). The method used was not specified for a total of 85 (5.9%) isolates among two studies [7,8].

The articles we found were from 13 countries and covered a 27-year collection period (1994–2021). Fosfomycin was tested alone for 1258 isolates (87%) and in combination with other antimicrobials for 187 isolates (13%). In vitro studies included 1413 isolates (97.8%), while in vivo studies included 32 isolates (2.2%).

#### 2.1.2. Mechanism of Resistance

Resistance to fosfomycin in *P. aeruginosa* primarily results from the overexpression of the activity of the inherent antibiotic-altering enzyme FosA or is caused by inactivation of the fosfomycin transport protein GlpT. FosA is a Mn(II)-dependent metalloenzyme that catalyzes the conjugation of glutathione to the epoxide ring of fosfomycin, inactivating the antibiotic. GlpT is the only fosfomycin transporter present in *P. aeruginosa*. Similar to FosA overexpression, a glpT mutation causes resistance to fosfomycin. Other acquired Fos enzymes (E, F, and H) have been associated with resistance to fosfomycin in *P. aeruginosa*. Among inherent resistance mechanisms, there are the peptidoglycan recycling enzymes (MupP, AmgK, and MurU), which help bypass fosfomycin-sensitive peptidoglycan de novo synthesis, and their upstream enzyme for muropeptide processing (NagZ, AmpD, and AnmK) [29]. Fosfomycin use is limited due to the lack of specific susceptibility breakpoints for *P. aeruginosa*. EUCAST does not publish breakpoints specific to *P. aeruginosa* but notes that wild-type isolates (isolates without resistance mechanisms for fosfomycin) were those with an ECOFF value of ≥256 mg/L. Among studies included in our review, no specific resistance mechanisms for fosfomycin have been deeply investigated.

In our statistical analysis (Table 2), 85 samples were not included because no MIC values were available [7,8]. A total of 1360 strains were analyzed, isolates were divided into two groups “Agar + G6P” (or traditional method), with a total of 1093 (80.4%) isolates, and “other methods” (i.e., broth microdilution, E-Test ECC), with a total of 267 (19.6%) isolates. Data from selected studies in our review show a total of 823 (60.5%) susceptible isolates with MIC values of≤ 128 mg/L (MIC range 1–128 mg/L) and 537 (39.5%) resistant isolates with MIC values of≥ 256 mg/L (MIC range 256–1024 mg/L). We found a statistically significant difference among isolates with MIC ≤ 128 mg/L and MIC ≥ 256 mg/L, between two groups, *p* < 0.00001. Considering the difference we reported among MICs values, let us suggest following EUCAST and CLSI recommendations to avoid false fosfomycin sensibility test results.

#### 2.1.3. Epidemiology

In recent years, the inappropriate use of antibiotics has led to the development of new resistance to antimicrobials; then, it is crucial to license new drugs that, in monotherapy or in combination, could be effective against MDR *P. aeruginosa* (MDR-PA) considering the high rates of morbidity and mortality.

As a matter of fact, in addition to intrinsic resistance to various antibiotics, the acquisition of resistance mechanisms through chromosomal mutations or the production of biofilms has enabled *P. aeruginosa* to escape the mechanisms of action of the most available drugs. According to the World Health Organisation (WHO), carbapenem-resistant *P. aeruginosa* (CPA) strains represent a “critical” pathogen needing new therapies. In addition, MDR-PA has been considered a very serious infection by the Centers for Disease Control and Prevention (CDC) over the past ten years, causing at least 32,600 cases, 2700 deaths, and $767 million in attributable healthcare costs each year [30].

Active surveillance of antibiotic resistance represents a valid tool to fight the spread of this phenomenon worldwide. In fact, according to the latest ISS (Istituto Superiore di Sanità) reports about the spread of MDR-PA in Italy, in 2020, among Gram-negative bacteria, 12.5% of *P. aeruginosa* isolates resulted in resistance to three or more antibiotics, including piperacillin-tazobactam, ceftazidime, carbapenems, aminoglycosides, and fluoroquinolones. In a study conducted by Riaño-Moreno et al. [9] on the distribution and resistance rates of MDR-PA in Europe, antimicrobial resistance rates are higher in low- and middle-income countries than in high-income countries. Although this effect has been related to lower community consumption of antibiotics in high-income countries compared to lower-middle-income countries, this could also be related to differences in national health policies, as the control of antimicrobial resistance is generally centralized in policies with national initiatives and commitments [31].

Globally, carbapenem-resistance due to Metallo-β-lactamase (MBL) production appears to be common in *P. aeruginosa*, with important implications about the choice of treatment options, as most β-lactamase inhibitors are unable to inhibit them. According to the available literature, fosfomycin is active against most cabapenem-resistant *Enterobacteriaceae* (CRE) and CPA. A potential new strategy to avoid problems with the development of resistance during fosfomycin monotherapy is to use a combination of antibiotics, especially for infections with a high bacterial load [32].

Another important mechanism is the production of OXA-48 mutations. According to CDC data, OXA-48-producing CPAs were detected in only 43 patients from 19 states in 2015. In contrast, they are commonly found in Europe, especially Mediterranean countries. OXA-48-like β-lactamases are notoriously difficult to detect in the clinical laboratory, causing the need to implement infection control measures. Since 2010, the prevalence of nosocomial outbreaks has been described worldwide, leading to endemic diffusion of OXA-48 strains largely distributed between Eurasia and Africa. It is important to underline that, over the last 20 years, OXA-48 and ‘OXA-48-like’ enzymes have proliferated to become the most widespread enterobacterial carbapenemases in much of Europe, North Africa, and the Middle East [33].

The attempt to find new therapeutic strategies against *P. aeruginosa* infections is gaining more and more interest over time. Of importance, the most important intrinsic resistance mechanism in *Pseudomonas* strains is given by the mechanisms of membrane waterproofing, downregulation of porins (mainly OprD) and overexpression of efflux pumps, all enhanced by overproduction of intrinsic AmpC. The aminopenicillins and cephalosporins (especially cefoxitin) are strong inducers of AmpC, and this can lead to overexpression of this enzyme, which accounts for resistance to many β-lactams, partly excluding cefepime, ceftolozane/tazobactam, and imipenem. In addition to overproduction, AmpC mutation can also occur, leading to resistance to both ceftolozane/tazobactam and ceftazidime/avibactam [34].

Of interest, *P. aeruginosa* is a major cause of life-threatening nosocomial infections in immunocompromised patients. As reported above, the main cause of cephalosporin resistance in *P. aeruginosa* is the overexpression of the chromosomal enzyme AmpC (mainly resistant to ceftazidime) and the production of MBL (resistant to cephalosporins and carbapenems). However, *P. aeruginosa*-producing, extended-spectrum β-lactamases (ESBLs) are often isolated [35]. According to Horcajada et al. [36], recently, an increase in *P. aeruginosa* MDR and extensive drug-resistant (XDR) strains has been observed, with rates between 15% and 30% in some geographical areas. Most European countries report resistance rates above 10% for all antimicrobial groups under surveillance. The use of fosfomycin would appear to be effective, especially in combination with new antimicrobials such as ceftazidime-avibactam or ceftolozane-tazobactam. Further studies and clinical series are needed to define the future role of fosfomycin in these infections, including the optimal dose and possible combinations.

### 2.2. Pharmacokinetic/Pharmacodynamic Relationship

According to preclinical evidence, the free area under the concentration-to-time curve to minimum inhibitory concentration ratio (*f*AUC/MIC) was defined as the best pharmacokinetic/pharmacodynamic (PK/PD) target for fosfomycin efficacy in infections caused by *P. aeruginosa* [37]. In a neutropenic murine thigh infection model against two *P. aeruginosa* strains, Lepak et al. [38] found that the AUC/MIC ratio was a very strong predictor of efficacy, with R^2^ equal to 0.92. Net stasis against the two *P. aeruginosa* strains was observed at AUC_0–24 h_/MIC ratio values of 11.3 and 17.9, respectively, whereas one-log kill was observed at AUC/MIC ratio values of 15.6 and 40.8 [38].

Similar to those observed for other antimicrobial agents (e.g., beta-lactams), more aggressive PK/PD targets are also required for fosfomycin in order to maximize both clinical efficacy and suppression of resistance emergence [39]. Specifically, a dynamic in vitro model found that an *f*AUC/MIC ratio of 489–1024 was required for suppressing fosfomycin resistance emergence against two MDR *P. aeruginosa* isolates, although these targets could not be achieved even with fosfomycin exposures well above those that can be safely achieved clinically [40]. These fosfomycin PK/PD targets were consistent with those previously identified in an in vitro dynamic hollow-fiber infection model study performed by Docobo-Pérez et al., reporting an AUC_0–24 h_/MIC ratio of≥ 3136 for suppressing resistance emergence against a susceptible CTX-M-15-producing *Escherichia coli* strain with a MIC of 1 mg/L [41].

It is noteworthy that studies assessing the relationship between the attainment of optimal fosfomycin PK/PD targets reported in preclinical evidence and clinical outcomes are currently limited. In a case series, including six patients affected by severe difficult-to-treat resistant *P. aeruginosa* (DTR-PA) (fosfomycin MIC range: 32–256 mg/L) bloodstream infection or pneumonia treated with continuous infusion (CI) ceftazidime-avibactam or extended infusion cefiderocol combined with CI fosfomycin during ceftolozane-tazobactam shortage, microbiological eradication was documented in four out of the four cases attaining optimal joint PK/PD targets, whereas only one out of the two patients attaining quasi-optimal joint PK/PD targets had a favorable microbiological outcome [13]. Specifically, the optimal joint PK/PD target was defined as the concomitant achievement of a fosfomycin AUC/MIC ratio > 40.8 and a ceftazidime steady-state concentration (C_ss_)/MIC ratio ≥ 4 coupled with avibactam C_ss_ > 4 mg/L or a cefiderocol trough concentration (C_min_)/MIC ratio ≥ 4 [13]. Similarly, clinical cure and microbiological eradication were documented in a case of postneurosurgical ventriculitis due to DTR *P. aeruginosa* (fosfomycin MIC equal to 64 mg/L) treated with CI ceftazidime-avibactam and fosfomycin according to a real-time optimization of PK/PD target attainment at the infection site [42]. Notably, the administration of fosfomycin 24 g/day by CI after a loading dose of 8 g allowed us to achieve a *f*AUC/MIC ratio in cerebrospinal fluid (CSF) ranging from 104.63 to 126.38 during overall treatment (above the desired threshold of 40.8), accounting for a CSF-to-plasma ratio of 0.42–0.5 [42]. Studies assessing fosfomycin penetration in different sites of infection are reported in Table 3.

### 2.3. Clinical Data

#### 2.3.1. Pneumonia

*P. aeruginosa* is a common cause of healthcare-associated pneumonia (HAP), including ventilator-associated pneumonia (VAP) [50]. Worldwide MDR and XDR *P. aeruginosa* are a growing threat. The antimicrobial treatment of patients with pneumonia due to MDR *P. aeruginosa* is challenging, and MDR *P. aeruginosa* clones cause increasing costs and worse outcomes [30]. In the presence of carbapenem-susceptible *P. aeruginosa* isolates, studies using the hollow-fiber infection model proposed a combination treatment with fosfomycin and carbapenem to enhance bacterial killing and reduce the emergence of antimicrobial resistance [51]. Similarly, the combination of fosfomycin with novel antimicrobials, such as ceftazidime/avibactam, ceftolozane/tazobactam or cefiderocol, has been proposed to treat patients with pneumonia due to CPA [30,52]. The combination of fosfomycin and ceftolozane/tazobactam against *P. aeruginosa* was reported to be synergic in studies in vitro, leading to ceftolozane/tazobactam MIC reduction [10,11,26].

Promising data were also reported from in vitro studies evaluating the combination of ceftazidime/avibactam and fosfomycin against *P. aeruginosa* [3,53]. However, clinical studies reporting on the use of antimicrobial combinations, including fosfomycin, for the treatment of patients with pneumonia are scarce.

A retrospective study was performed in a tertiary Italian hospital to analyze the efficacy and safety of fosfomycin in a real-life setting [54]. Overall, the study included 343 adult patients, including 63 patients with HAP. Of the 343 patients, 42 (12%) had an infection due to *P. aeruginosa* [54]. However, the study included only six patients with infection due to *P. aeruginosa* treated with a combination of fosfomycin and ceftazidime/avibactam, and death occurred in all these six patients [54].

A retrospective study performed in two Italian intensive care units reported on 23 COVID-19 patients with VAP [55]. The study included eight patients with pneumonia due to *P. aeruginosa* treated with a combination of ceftazidime/avibactam and fosfomycin. Among these eight patients, four survived at 30 days, with a 50% mortality rate at 30 days [55]. Recently, a retrospective study reported on the use of a combination treatment, including fosfomycin, for patients with pneumonia due to MDR *P. aeruginosa* [13]. The study described three patients treated with cefiderocol and fosfomycin, all of them survived at 30 days. The study also included two patients treated with ceftazidime/avibactam and fosfomycin, reporting a negative outcome at 30 days [13].

Future studies are needed to optimize the algorithms and the dosage of a combination therapy with intravenous fosfomycin for the treatment of patients with pneumonia due to MDR *P. aeruginosa* [56].

#### 2.3.2. Bone and Prosthetic Joint Infections

Worldwide, bone and joint infections represent a major cause of morbidity and disability [57]. Gram-positive bacteria are the most common isolated pathogens causing bone and joint infections, even if the prevalence of Gram-negative bacteria is increasing [58].

The management of bone and joint infections, including prosthetic joint infections, is often challenging due to the frequent need for orthopedic surgery, the reduced bone tissue penetration of many antibiotics, and the increasing rate of MDR bacteria. Bone infections can cause hypoxia and abscess formation in the bone tissue, two conditions that contribute to the decreased efficacy of antibiotics [57]. Fosfomycin possesses a favorable PK/PD profile and retains activity in acid and hypoxic conditions [49,59,60]. Moreover, studies report a synergistic effect of fosfomycin and fluoroquinolones against biofilm-forming *P. aeruginosa*, even though research is lacking [61,62]. However, it is important to keep in mind that the activity of fosfomycin may decrease in the presence of a high bacterial burden in the bone tissue [63]. The use of fosfomycin should always be considered in combination with other antibiotics to take advantage of the synergistic effect and to reduce the risk of resistance development [63]. Therefore, fosfomycin should be considered for the antibiotic combination treatment of bone and joint infections, including infections due to *P. aeruginosa* [64].

Clinical studies reporting on the use of fosfomycin for the treatment of patients with bone infections are scarce.

A retrospective study on pediatric patients included 103 patients with acute hematogenous osteomyelitis. In this study, hematogenous osteomyelitis were mainly caused by *S. aureus*. The included patients received treatment with fosfomycin in monotherapy (n: 23), fosfomycin in combination with a beta-lactam (n: 47) or other antibiotic regimens not including fosfomycin (n: 33).

No differences in the outcome were observed among the three patient groups, with percentages of remission achieved in 100%, 98%, and 97% of the patients, respectively [65].

More recently, a case report was published on a prosthetic joint infection due to MDR *S. epidermidis* successfully treated with debridement and combination antibiotic therapy with daptomycin and fosfomycin, 8 g daily [66].

Regarding the available literature on the use of fosfomycin for the treatment of patients with bone infections due to *P. aeruginosa*, the first study in chronological order is a prospective clinical trial performed in 1989, to evaluate the effectiveness of an antibiotic combination therapy including fosfomycin. Fosfomycin was administered at a dosage of 5 g every 8 h, with a loading dose of 5 or 10 g. The trial enrolled 60 patients with chronic, posttraumatic osteomyelitis. The osteomyelitis was predominantly located in the tibia (43 patients) and the femur (13 patients). In the study, *P. aeruginosa* accounted for 16.7% of the pathogens isolated. The mean MIC_90_ value of the *P. aeruginosa* isolates was 64 mg/L. Overall, after a mean follow-up of 37 months, the authors reported an outcome defined as “excellent” in 54.7% of the included patients, “remissions” in 18.9%, and “treatment failure” in 26.4% [67]. More recently, Wong et al. reported a case of osteomyelitis caused by MDR *P. aeruginosa* treated with fosfomycin in combination with ceftolozane/tazobactam and then meropenem. After a 14-day course of intravenous fosfomycin, the authors reported an improvement of the patient’s wound [68]. Moreover, Narayanasamy et al. reported a successful combination treatment with fosfomycin (16 g a day) and colistin for a patient with osteomyelitis due to XDR *P. aeruginosa* [69].

Future research is needed to further optimize the combination therapy with intravenous fosfomycin for the treatment of patients with bone and joint infections.

#### 2.3.3. Urinary Tract Infections

Fosfomycin is widely used for urinary tract infection (UTI) due to its high concentration in urine, especially in cases of low pH [70]. The use of fosfomycin in UTI dates back several years, especially the oral formulation for uncomplicated cystitis in women showed similar efficacy in comparison to standard of care [71,72,73,74,75]. However, fewer studies evaluated the use of fosfomycin for urinary tract infections caused by MDR pathogens, including *P. aeruginosa*. For example, only one case report showed the efficacy of 3 weeks of intravenous fosfomycin plus aztreonam for bla_VIM-2_ *P. aeruginosa* prostatitis [76].

The randomized clinical trial by Kaye et al. [77] compared i.v. fosfomycin 6 g every 8 h versus piperacillin/tazobactam 4.5 g every 8 h for complicated UTIs (cUTI), including pyelonephritis, in patients admitted to hospital and randomized 1:1 to receive either fosfomycin or piperacillin/tazobactam. Of 465 patients enrolled, 233 were treated with fosfomycin and 231 with piperacillin/tazobactam. The study showed a similar clinical cure rate between fosfomycin and piperacillin/tazobactam (90.8% vs. 91.6%, respectively). Among the population observed, *P. aeruginosa* was involved in 4.3% of patients among the fosfomycin group and in 5.1% of patients among the piperacillin/tazobactam group; all those patients reached clinical cure despite a low microbiological cure rate in both groups (37.5% vs. 44.4%, respectively). The authors concluded that i.v. fosfomycin could be an effective treatment for cUTIs, including pyelonephritis.

An observational retrospective study by Neurer et al. [78] evaluated the microbiological cure rate among 41 hospitalized patients with cystitis due to MDR pathogens treated with fosfomycin trometamol. *P. aeruginosa* was found in eight urine cultures, 6/8 (75%) of isolates were susceptible, with a MIC_50_ 8 mg/mL and a MIC_90_ 128 mg/mL. The authors found that solid organ transplantation and ureteral stents were associated with microbiological failure in the overall population. Microbiological cure was reached in 3/8 (38%) of *P. aeruginosa* UTIs. One patient (liver transplanted) with microbiological failure developed fosfomycin resistance; the authors hypothesized that higher MIC values may have contributed to the microbiological failure, whereas fosfomycin resistance occurs without a fitness cost in *P. aeruginosa*, as suggested by other authors [79]. This statement could not explain high clinical cures despite variable resistance rates, as shown by the following studies [80,81].

Another observational study by Zhanel et al. [80] was conducted among 59 hospitalized patients who received intravenous fosfomycin. Among 59 patients, two patients were treated for MDR *P. aeruginosa* cUTI. Both patients were treated with 4 g every 8 h of fosfomycin and reached clinical and microbiological cures.

The observational study by Dinh et al. [81] evaluated 116 hospitalized patients receiving intravenous fosfomycin in association with other antibiotics (mainly aminoglycoside and beta-lactams). The study was conducted retrospectively, analyzing the clinical records. Among the overall population, 33/116 had a *P. aeruginosa* infection, with 27/33 (82%) MDR *P. aeruginosa*. Among them, 7/27 (26%) had a *P. aeruginosa* UTI, 4/7 in association with ceftazidime and 3/7 with aminoglycoside. The dosage of fosfomycin was different among patients (mainly 12 g/day, only one case 16 g/day and one case 8 g/day), and all patients reached microbiological and clinical cures.

Giancola et al. [82] performed an observational study among 57 hospitalized patients with UTI who received ≥ 1 dose of fosfomycin. Among the overall population, cUTI was observed in 44/57 (77%). *P. aeruginosa* was responsible for 8/57 (14%) of cases, all classified as cUTI, and 6/8 (75%) were MDR. Data of patients divided by microorganisms were not available. Clinical information was available only for 27/57 patients, and despite an overall clinical cure of 96% (26/27), the only information among the eight *P. aeruginosa-*infected patients is that one patient had a reinfection, one patient had a clinical failure. The high rate of MDR *P. aeruginosa* (75%) and the presence of cUTI among all *P. aeruginosa*-infected patients, lacking microbiological and clinical data, leaves us free to speculate on a possible high clinical failure rate due to predisposing risk factors.

To conclude, available literature data among MDR *P. aeruginosa* UTIs showed a high clinical cure rate (55–100%) and variable microbiological cure rates (38–100%) despite a high fosfomycin resistance rate among MDR *P. aeruginosa* (50–90%). Fosfomycin is confirmed as a valid therapeutic choice, especially in combination with aminoglycoside, carbapenem, ceftazidime, and aztreonam. The high clinical cure rate and the variable microbiological cure rate may be due to the lack of universally accepted MIC breakpoints for fosfomycin.

#### 2.3.4. Bloodstream Infections

*P. aeruginosa* is a major cause of bloodstream infections (BSI) associated with high mortality, ranking third, after *Escherichia coli* and *Klebsiella* spp., among Gram-negative bacteria isolated during nosocomial BSI, and seventh among all pathogens [83]. Up to 90% of patients who present with *P. aeruginosa* BSI have a severe underlying disease, most often malignancy, or a chronic disease, such as diabetes, renal failure, cirrhosis, heart failure, chronic pancreatitis, chronic obstructive pulmonary disease, solid organ transplantation, or acquired immunodeficiency syndrome [83]. Adequate empirical antimicrobial therapy, waiting to receive the antibiogram, seems to improve survival, but previously used antimicrobial agents should be avoided [83]. Some authors recommend combining fosfomycin with other antibiotics for *P. aeruginosa* [84]. This recommendation arises from the rising rates of bacterial resistance to antibiotics worldwide [85]. Fewer studies evaluated the use of fosfomycin for BSI caused by *P. aeruginosa*.

An observational retrospective study by Tumbarello et al. [86] evaluated, by a case-case-control study of 106 patients with *P. aeruginosa* BSI, risk factors for the isolation of MDR and non-MDR *P. aeruginosa* in blood cultures. The authors found that the presence of a central venous catheter (CVC), previous antibiotic therapy, and corticosteroid therapy were independent risk factors for the isolation of MDR *P. aeruginosa*. Instead, previous bloodstream infection, neutrophil count <500/mm^3^, urinary catheterization, and the presence of CVC were independent risk factors for non-MDR *P. aeruginosa*. The overall 21-day mortality rate was 34%, and independent risk factors for mortality were septic shock, MDR *P. aeruginosa* infection, and inadequate initial antimicrobial therapy.

In a retrospective study of a case series, six patients with documented severe difficult-to-treat *P. aeruginosa* infections (HAP/VAP and/or BSI) were treated with continuous-infusion fosfomycin in combination with extended-infusion cefiderocol or continuous-infusion ceftazidime-avibactam. BSI was found in one case, and VAP plus BSI in two cases. All patients were treated with fosfomycin in combination with cefiderocol, achieving microbiological eradication. Authors found that the combination of fosfomycin with beta-lactams was superior to either drug alone for the treatment of MDR *P. aeruginosa* [13]. The fosfomycin resistance rate can be as high as 30%, and some authors report a 54% synergistic effect of fosfomycin in combination with beta-lactams (i.e., ceftazidime, meropenem, aztreonam, cefiderocol), quinolones, and aminoglycosides [87]. Intravenous fosfomycin represents a promising option in the treatment of MDR *P. aeruginosa* BSI, due to its high plasma concentrations, particularly in combination with aminoglycosides, carbapenems, and colistin [9].

#### 2.3.5. Central Nervous System

As stated above, fosfomycin is a very small hydrophilic compound with low molecular mass; notwithstanding its hydrophilicity, it crosses the blood-brain barrier entering the central nervous system (CNS) either in the presence or absence of meningeal inflammation more readily than beta-lactam antibiotics [88]. When meninges are uninflamed or only mildly inflamed, the ratio between the AUC in the CSF and the AUC in the serum is estimated to be around 0.2, much higher than cephalosporins and overlapping with carbapenems [89]. In the case of strong meningeal phlogosis, the levels of the drug in the CSF may even increase, according to old studies [90]. On the other hand, very high concentrations should be reached to achieve adequate bacterial killing in the CSF; therefore, fosfomycin monotherapy for CNS infection is discouraged [91]. According to expert opinion, it should be considered reserve antibiotic for *Staphylococcus aureus* and *P. aeruginosa* CNS infections in the context of targeted treatment [89]. Unfortunately, clinical experience from literature in this setting is scant. In particular, when focusing on DTR-PA infections of the CNS treated with fosfomycin, only two reports are present in the literature, all from Italy [42,92].

A case report by Frattari et al. showed successful treatment of intravenous fosfomycin in a breakthrough otogenous meningitis caused by extensively drug-resistant *P. aeruginosa* (XDR-PA) in a 27-year-old male patient treated with meropenem and colistin for XDR-PA BSI and pneumonia following a car-crash polytrauma [92]. Three days after the beginning of this regimen, the onset of neurological signs and symptoms prompted lumbar puncture, yielding a cloudy CSF with pleocitosis. In the light of a microbiological preliminary result of culture compatible with a *P. aeruginosa* CNS infection (stemming from a right middle-ear and mastoid suppurative infection), the caring physicians resorted to a salvage regimen based on high-dose ceftolozane/tazobactam (3 g q 8 h), high-dose fosfomycin (4 g q 6 h) and rifampicin (600 mg q 12 h). The new regimen was effective along with source control through mastoidectomy with tympanoplasty; fosfomycin was discontinued after 7 days, ceftolozane-tazobactam after 14 days. Repeated lumbar puncture documented CSF sterilization; the XDR-PA strain initially isolated from CSF was resistant to carbapenems in, the absence of carbapenemases production. Unfortunately, a MIC value for fosfomycin could not be obtained and no synergism test was carried out [92].

The second report describes two cases of postneurosurgical ventriculitis by carbapenem-resistant, Gram-negative pathogens, specifically *Klebsiella pneumoniae* and *P. aeruginosa*, in which treatment was optimized by means of a real-time clinical pharmacological advice program aiming at maximizing pharmacodynamic target attainment (PD-TA) at the infection site [42]. The latter case involved a 52-year-old male who had undergone neurosurgery owing to a pineal neoplasm complicated by obstructive hydrocephalus and positioned an external ventricular drain (EVD). Subsequently, he developed fever and headache relapse: a lumbar puncture was performed; the culture yielded a DTR-PA strain susceptible to ceftazidime/avibactam, amikacin, and ceftolozane/tazobactam. Notably, testing by agar dilution (the reference method) showed a MIC value for fosfomycin equal to 64 mg/L. After removing and substituting the infected EVD, empiric and not active antibiotic therapy was switched to targeted ceftazidime/avibactam (2.5 g/q 6 h over 6 h through continuous infusions after 2.5 g of loading dose) plus fosfomycin (8 g as loading dose followed by 16 g q 24 h through continuous infusion). Plasma and CSF therapeutic drug monitoring (TDM) of both agents was implemented; as far as fosfomycin was concerned, the desired target in the CSF was *f*AUC/MIC ratio ≥ 40.8 [38]. In the light of initial suboptimal PD-TA, progressive blood-brain barrier healing and high creatinine clearance (above 100 mL/min/1.73 m^2^), the dosages of both antibiotics were increased: up to 5 g/q 8 h over 8 h for ceftazidime/avibactam and 24 g/q 24 h for fosfomycin, both through continuous infusion. The clinical course was favorable after a 3-week course of therapy [42].

### 2.4. Proposed Schemes

In Table 4, we propose a therapeutic scheme for fosfomycin in association with other drugs for the treatment of MDR *P. aeruginosa*

## 3. Methods

To improve the reliability and the quality of this review, we followed the methodological recommendations provided by the Scale for the Assessment of Narrative Review Articles (SANRA) through the following six areas: an explanation of the review’s importance, definitions of the aims of the review, description of the literature search, appropriate referencing, scientific reasoning, and presentation of relevant and appropriate endpoint data [93]. Major details are provided in the Supplementary Material. Relevant articles written in English language were identified through PubMed screening up to 31 July 2023, by using a combination of appropriate keywords according to the topic. We performed a statistical analysis (Table 2) to evaluate the relationship between isolates with fosfomycin MIC values ≤ 128 mg/L or ≥ 256 mg/L among studies with different sensibility testing (Agar + glucose-6-phosphate vs. other methods). Analysis was performed with SPSS v29, and a chi-square test with Yates correction was used, with a *p*-value ≤ 0.05.

## 4. Conclusions

Growing evidence of intravenous fosfomycin use for *P. aeruginosa* infections is being reported. Microbiological issues exist since agar dilution (time-consuming) is the reference method, and EUCAST does not provide clinical breakpoints. From a pharmacological point of view, the AUC/MIC ratio is a strong predictor of efficacy, and the strategy of a loading dose followed by continuous infusion is becoming increasingly utilized. The optimum dosage is a matter of debate; however, in life-threatening infections, the administration of high doses (i.e., 24 g q 24 h) with TDM is reasonable. Given the common *Pseudomonas* heteroresistance for fosfomycin, the administration within a combination regimen appears prudent, is used by most clinicians, and is recommended by most experts. The drug has properties that make it appealing even for particular infections caused by *Pseudomonas*, such as those with biofilm production or involving the CNS. The evidence of microbiological and clinical efficacy is good for urinary tract infections and pulmonary infections and growing for infections of bone, prosthetic joints, bloodstream, and the CNS.

## Figures and Tables

**Table 1:** In vivo and in vitro studies evaluating fosfomycin susceptibility testing, alone, or in combination.

Ref.	Year	Country	Collection Period	Isolates— n (%)	Isolates with MIC ≤ 128 mg/L—n (%)	MIC Range for Fosfomycin (mg/L)	Method	Testing in Combination—n (%)	Antibiotic in Combination	Notes
[9]	2020	Italy	NA	62	60 (97%)	4–>256	Standard agar dilution + G6P	NA	NA	In vitro
[10]	2017	USA	2017	4	4 (100%)	16–≥64	NA	4 (100%)	Ceftolozane/Tazobactam	In vitro
[11]	2020	Brasil & USA	2019	27	12 (44%)	32–>1024	Etest VR gradient strips	27 (100%)	Ceftolozane/Tazobactam	In vitro
[12]	2013	Spain	2013	206	178 (86.4%)	2–≥1024	Standard agar dilution + G6P	NA	Na	In vitro
[13]	2022	Italy	2021	6	5 (83%)	32–>256	Standard agar dilution + G6P	6 (100%)	Cefiderocol 5 (97%); Ceftazidime/Avibactam 1 (3%)	In vivo
[14]	2008	Greece	2006–2007	30	27 (90%)	4–≥512	Standard agar dilution + G6P	NA	NA	In vitro
[7]	2007	France	1996–2004	59	NA	NA	NA	NA	NA	In vitro
[15]	2007	Japan	2004–2006	45	42 (93.3%)	NA	NA	NA	NA	In vitro
[16]	1997	France	1994–1995	40	37 (92.5%)	2–≥512	Standard agar diluition + G6P	40	Ceftazidime, Imipenem, Amikacin and Ciprofloxacin	In vitro
[17]	2002	Japan	1995–1998	30	20 (66.66%)	4–≥256	Broth microdilution + G6P	30	Cefepime, Aztreonam, Meropenem, Imipenem, Ceftazidime, Gentamicin, Piperacillin, Levofloxacin	In vitro
[18]	1998	Germany	1996–1997	210	4 (1.9%)	NA	Standard agar dilution + G6P	2	Rifampin, Amikacin	In vitro
[19]	1996	France	NA	214	95 (44.4%)	≤8–≥128	Standard agar dilution + G6P	12	Ceftazidime	In vitro
[8]	2008	UK	2005–2008	26	NA	NA	NA	26	Colistin, Ciprfloxacin, Piperacillin/Tazobactam, Tobramicin	In vivo
[20]	2016	Spain	NA	47	47 (100%)	2–128	Agar + Etest strips	NA	NA	In vitro
[21]	2018	Canada	2005–2013	24	18 (75%)	2–>1024	Broth microdilution + G6P	NA	NA	In vitro
[22]	2019	India	2016	32	16 (50%)	16–>1024	Standard agar dilution + G6P	NA	NA	In vitro
[23]	2021	Italy	2019	38	33 (87%)	2–≥128	Standard agar dilution + G6P	NA	NA	In vitro
[24]	2019	Brazil	NA	19	15 (79%)	32–>512	NA	19 (100%)	Meropenem	In vitro
[25]	2020	Egypt	2018	50	29 (58%)	2–>128	Agar + Etest strips	NA	NA	In vitro
[26]	2019	USA	NA	21	10 (48%)	4–>512	Broth microdilution	21 (100%)	Ceftazidime-Avibactam, Amikacin, Aztreonam, Colistin, Meropenem	In vitro
[27]	2022	Netherland	NA	57	53 (93%)	1–>256	Standard agar dilution + G6P	NA	NA	In vitro
[28]	2020	USA	2013	198	118 (60%)	1–>256	Standard agar dilution + G6P	NA	NA	In vitro

Ref: reference. MIC: minimum inhibitory concentration. G6P: glucose-6-phosphate. NA: not available.

**Table 2:** Statistical analysis of fosfomycin MIC values among studies with different sensibility testing (Agar + G6P vs. other methods).

	Agar + G6P	Other Methods	Total	*p*-Value
MIC ≤ 128 mg/L–n (%)	626 (46%)	197 (14.5%) †	823 (60.5%)	*p* < 0.00001
MIC ≥ 256 mg/L–n (%)	467 (34.4%)	70 (5.1%) †	537 (39.5%)	
Isolates–n (%)	1093 (80.4%)	267 (19.6%) †	1360 (100%) †	

The chi-square statistic with Yates correction is 23.7904, *p*-value is < 0.00001. MIC: minimum inhibitory concentration. G6P: glucose-6-phosphate. † MIC not available for 85 samples out of 1445 overall isolates.

**Table 3:** Fosfomycin penetration and assessment of PK/PD target attainment in different sites of infection.

Site of Infection	Dose	Absolute Concentrations	Penetration Rate (AUC_tissue_/AUC_plasma_)	PK/PD Target Attainment	References
Lung	4 g single dose	AUC 1221 mg × h/L	0.53 ± 0.31	Up to MIC of 16 mg/L	[43]
CNS	24 g/day CI 8 g q 8 h over a 30-min infusion	Median C_ss_ 104 mg/L (IQR 65–269 mg/L) Median AUC 2381 mg × h/L (IQR 1585–3456 mg × h/L) AUC 885 mg × h/L	0.46 (IQR 0.36–0.59) 0.27 ± 0.08	Up to MIC of 32 mg/L Up to MIC of 16 mg/L	[44] [45]
Muscle	8 g single dose	Median AUC_0–4_ 477 mg × h/L (IQR 226–860 mg × h/L) AUC_0–24_ 2862 mg/L	0.71 (IQR 0.34–1.05)	Up to MIC of 64 mg/L	[46]
Subcutaneous tissue	15.5 g ± 3.9 g/day in three doses over a 30-min infusion	AUC_0–24_ 2346 mg/L	0.60–0.73	Up to MIC of 32 mg/L	[47]
Abdominal abscess	8 g single dose	Mean C_ss_ 162 ± 64 mg/L AUC_0–24_ 986 mg/L	0.42	Up to MIC of 16 mg/L	[48]
Bone	100 mg/kg/day	C_max_ 96.4 ± 14.5 mg/kg AUC_0–12_ 511.0 ± 100.7 mg∙h/kg	0.43	Up to MIC of 16 mg/L	[49]
Plasma	24 g/day CI	AUC_0–24_ 4800 mg × h/L (IQR 3816–7152 mg × h/L)	-	Up to MIC of 64 mg/L	[44]

AUC: area under concentration-to-time curve; CI: continuous infusion; CNS: central nervous system; C_ss_: steady-state concentrations; IQR: interquartile range; MIC: minimum inhibitory concentration; PK/PD: pharmacokinetic/pharmacodynamic.

**Table 4:** Empirical association therapy for fosfomycin in case of *P. aeruginosa* MDR infection, suggested by available literature data and expert opinion. In the case of MIC 64–128, consider using 24 g/4 h of fosfomycin. The total fosfomycin daily dose could be divided into three or four daily administrations or in a continuous infusion.

	Lung Infections	Bone/PJI	UTI	CNS Infections	BSI
MIC < 16	15–18 g/24 h	15 g/24 h	12 g/24 h	18 g/24 h	18 g/24 h
MIC 16–32	24 g/24 h (HAP/VAP)	24 g/24 h	18 g/24 h	24 g/24 h	24 g/24 h
Combination suggested	Ceftolozane/Tazobactam Meropenem or Imipenem Levofloxacin	Guided by microbiological culture. Remove infected prosthesis.	Aminoglycoside Cefepime Ceftolozane/Tazobactam Meropenem or Imipenem Colistin	Meropenem or Imipenem Aztreonam * Quinolones Ceftolozane/Tazobactam *	Aminoglycoside, Cefepime, Ceftazidime, Ceftolozane/Tazobactam, Meropenem or Imipenem, Colistin,
Alternative	Cefepime, Ceftazidime Piperacillin/Tazobactam Ceftazidime/Avibactam Aztreonam * Colistin * Aminoglycoside *	Hemodynamical instable: Levofloxacin Cefepime, Ceftazidime Ceftolozane/Tazobactam Tigecycline Meropenem Aztreonam	Ceftazidime Piperacillin/Tazobactam Ceftazidime/Avibactam Aztreonam Quinolones	Remove the device, if any. Evaluate co-administration of intrathecal Aminoglycoside or Colistin	Piperacillin/Tazobactam Aztreonam Ceftazidime/Avibactam

Aminoglycoside: amikacin or tobramycin is preferred over gentamicin. BSI: Bloodstream Infections; CNS: central nervous system; HAP: hospital-acquired pneumonia; MDR: multidrug resistance; PJI: prosthetic joint infection; UTI: urinary tract infections. VAP: ventilator-acquired pneumonia; XDR: extensive drug resistance. * Low level in epithelial lining fluid or in cerebral spinal fluid. Note: Considering the risk of resistance induced by overuse and inappropriate use, novel anti-Gram-negative MDR drugs (i.e., Cefiderocol and Imipenem/Relebactam etc.) should be sparing and used empirically in selected cases: i.e., septic shock or hemodynamically unstable AND local data showed a high percentage of XDR microorganism or previously isolated microorganism resistant to meropenem, colistin and/or aminoglycosides. A previous patient’s antibiogram or local microbiological data could guide clinicians.

## Data Availability

Not applicable.

## Supplementary Materials

The following supporting information can be downloaded at https://www.mdpi.com/article/10.3390/antibiotics12121653/s1, Table S1: SANRA Quality Items. Reference [94] is cited in the supplementary materials.
