# Supplementary material for: Intravenous Fosfomycin for Systemic Multidrug-Resistant Pseudomonas aeruginosa Infections
Source: Antibiotics (Basel). 2023 Nov 23;12(12):1653. doi: 10.3390/antibiotics12121653 (PMC10741068; doi:10.3390/antibiotics12121653)
Supplement: Supplementary file 1 [file antibiotics-12-01653-s001.zip › antibiotics-2671989-supplementary.pdf]

Supplementary Material: Methods

We performed a narrative review, defined as a review of published materials that examine the currently available literature [94]. Its goals are the following: to summarize previous work, avoid duplication, and identify potential research or literature gaps. To maximize its reliability and quality, the narrative review was conceived, developed, and reported considering the six items suggested by the Scale for the Assessment of Narrative Review Articles (SANRA; Table S1) [93].

Table S1. Quality Items (SANRA).

Item	Implementation
Justification of the article's importance for its readership	Outlining the clinical problem and how the review addresses eventual gaps.
Statement of concrete aims	Providing specific aims of the review (addressing microbiological, pharmacological, and clinical data).
Description of the literature search	Brief description of literature search.
Referencing	Present references to justify all key statements.
Scientific reasoning	Supporting main arguments with evidence, clarifying the type and levels of evidence that exist for each argument.
Appropriate presentation of data	Giving accurate data relevant to each argument.
